# Myasthenic Crisis Precipitated by Raoultella Planticola in an Immunocompromised Host

**DOI:** 10.7759/cureus.22335

**Published:** 2022-02-17

**Authors:** Yashvir Rugbeer, Juan Jansen van Vuuren, Ansuya K Naidoo, Neil Naidoo

**Affiliations:** 1 Department of Neurology, Grey's Hospital, Pietermaritzburg, ZAF; 2 Clinical Medicine, University of KwaZulu-Natal, Pietermaritzburg, ZAF; 3 Department of Neurology, University of KwaZulu-Natal, Pietermaritzburg, ZAF

**Keywords:** myasthenia gravis, lower respiratory tract infection, gram negative bacteria, neuromuscular junction, raoultella planticola, myasthenic crisis

## Abstract

We present a case of a 39-year-old male patient who was previously diagnosed with myasthenia gravis. He presented in a myasthenic crisis secondary to a lower respiratory tract infection, with the implicated organism being *Raoultella planticola. *He was referred to the intensive care unit (ICU) and required ventilatory support due to respiratory insufficiency. Early broad-spectrum antibiotics for a suspected bacterial infection were provided in combination with management specific to the myasthenic crisis. The patient made a full recovery and has displayed a good clinical response. This case report explores his presentation and aims to provide further literature on the incidence and description of *R. planticola.*

## Introduction

Myasthenia gravis (MG) is the most common primary disorder of neuromuscular junction (NMJ) transmission [1]. The pathophysiological process results from the binding of antibodies to acetylcholine receptors, which results in failure of neuromuscular transmission and reduced muscle action potentials [1]. Patients present with variable effort-induced weakness of select muscle groups, depending on the specific MG subtype [1]. Diagnosis is reliant on history and clinical examination in addition to various diagnostic tests such as confirmatory antibodies (i.e., acetylcholine receptor antibodies and antimuscle-specific kinase antibodies), repetitive nerve stimulation, and single fiber electromyography [1]. A multitude of treatment options, symptomatic and immunomodulatory, are available, which prove to be effective with minimal medium and long-term morbidity [1].

A myasthenic crisis is defined as a significant worsening of myasthenic-type weakness, requiring advanced ventilator support as a result of respiratory failure [2,3]. Patients typically present with generalized weakness or prominent bulbar symptoms. Precipitants include alterations in MG-specific treatments, concomitant use of certain medications, and physiological stressors, such as surgery or infection, with the latter being a frequent trigger [2,3]. A case series in New York noted infection in 38% of patients presenting with myasthenic crisis. Bacterial pneumonia was the most frequent association, followed by a bacterial or viral upper respiratory tract infection [4].

## Case presentation

A 39-year-old male patient, known to be HIV-positive virologically suppressed with a CD4 count of 599 cells/µL, was diagnosed with MG following a history of progressively worsening diplopia, dysphagia, muscle weakness, and fatiguability. Table 1 summarizes his relevant clinical findings on the initial presentation. Nerve conduction studies illustrated a decremental response of compound muscle action potentials (CMAPs) following repetitive nerve stimulation, confirming a disorder of neuromuscular transmission. Serum acetylcholine receptor antibodies were initially negative but were equivocal with a value of 0.44 nmol/L on repeat testing. The patient was placed on pyridostigmine 60 mg eight-hourly, prednisone 5 mg daily with weekly increments of 5 mg, and azathioprine 50 mg daily.

**Table 1:** Clinical findings on presentation

Category	Clinical Findings
Higher functions	Intact
Meningism	Nil
Cranial nerves	Bilateral ptosis (right eye: 40%, left eye: 20%), absent bilateral eye elevation and depression, right eye abduction and adduction movements of 20%, left eye abduction movement of 90% and adduction movement of 10%, no eye convergence, neck flexion graded at 4+, positive Cogan’s lid twitch sign, positive ice-pack test - right eye: 10% ptosis (improved from 40%), left eye: no ptosis (improved from 20%)
Motor system	Wasting of bilateral intrinsic hand muscles and bilateral extensor digitorum brevis muscles, reduced tone in upper and lower limbs, muscle fatiguability demonstrated on exercise, and globally areflexic
Sensory system	Reduced sensation to pain and temperature in a glove and stocking distribution (likely an HIV-associated peripheral neuropathy)
Co-ordination	Intact
Gait	Normal

Ten days later, the patient presented to the emergency unit with acute deterioration of his condition. The patient reported a three-day history of a productive cough, fever, and malaise, with worsening weakness and increased respiratory effort. Clinical examination revealed sinus tachycardia and a tachypnoea of 40 breaths per minute. Chest expansion was reduced with coarse crepitations over the left lung fields. Bilateral ptosis with complete opthalmoplegia and poor bulbar function was seen. Motor examination revealed distal muscle weakness with globally reduced tone and absent reflexes.

**Figure 1:**
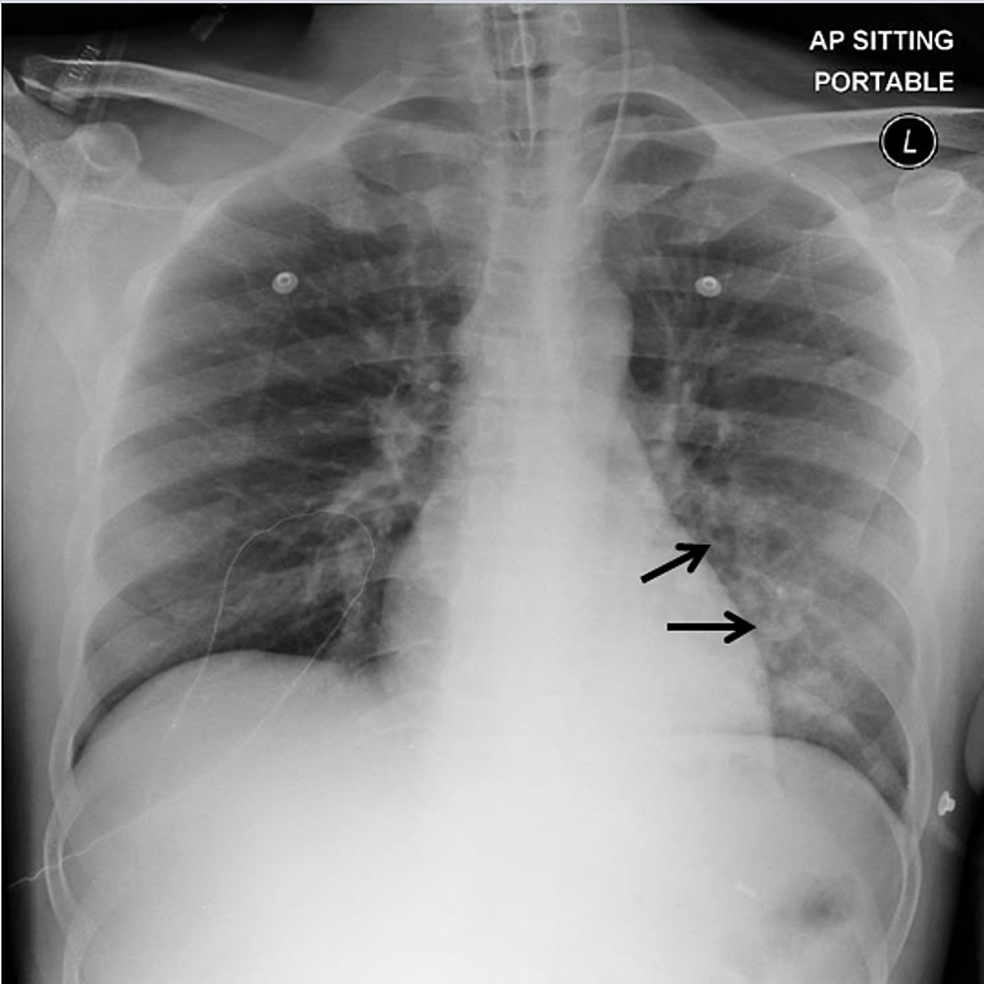
Chest x-ray of the patient The image shows a chest x-ray of the patient taken upon admission to the ICU. The arrows illustrate patchy infiltrates of the left lower lung fields. A diagnosis of a lower respiratory tract infection was made, secondary to bacterial pneumonia.

The patient was referred to the intensive care unit (ICU) for airway and respiratory support, including intubation and ventilation for four days, and was treated with intravenous neostigmine at 2 mg/hour together with high-dose prednisone at 60 mg daily and intravenous immunoglobulin at 0.4 g/kg for five days. Piperacillin/tazobactam was given empirically at 4.5 g intravenous infusion (IVI) six-hourly for a suspected lower respiratory tract infection (Figure 1).

His endotracheal aspirate cultured the organism *Raoultella planticola*. Laboratory investigations revealed an elevated white cell count of 14.9 cells x 10^9^/L with an absolute neutrophil count of 8.1 cells x 10^9^/L, a C-reactive protein of 22 mg/L, and a procalcitonin of 0.01 μg/L. He made a good recovery and was later discharged to a general medical ward, where he received ongoing rehabilitation and care. He was later discharged on a higher dose of pyridostigmine and azathioprine and has displayed a good clinical response on follow-up.

## Discussion

Myasthenic crises may be the index presentation of MG or can present as an exacerbation of a previously well patient's condition [1]. Myasthenic crises carry an in-hospital mortality rate of 4.47% compared to a mortality rate of 2.2% in MG [4]; therefore, prompt identification and management of the precipitant are required. MG in HIV-infected patients should be managed in a manner similar to HIV-uninfected patients, with an emphasis on effective antiretroviral therapy [5]. A lower than detectable HIV viral load should be evident prior to the initiation of immunosuppressive therapies, which should be administered based upon the severity of MG [5]. Our patient presented with features suggestive of a lower respiratory tract infection and required early invasive respiratory support. Based on ancillary investigations, a provisional diagnosis of bacterial infection was made, and early broad-spectrum antibiotics were initiated.

*R. planticola *is a gram-negative bacterium of the genus *Raoultella *[6]. It is a ubiquitous, non-motile, and encapsulated rod-shaped organism that is most frequently found in water and soil [6,7]. It characteristically inhabits the gastrointestinal tract and upper respiratory tract and has been implicated as the causative agent in pneumonia, biliary tract infections, bacteremia, and, most recently, urinary tract infections [7]. It has been found to cause infection in humans quite infrequently, with minimal literature of its incidence published. Although uncommon, this organism is increasingly being described as pathogenic, particularly in immunocompromised patients [6,7]. Other risk factors are invasive medical procedures, consumption of seafood, and being exposed to contaminated water or soil [8].

The use of high-dose corticosteroids along with the patient’s positive HIV status likely lessened his immune response, predisposing him to contract community-acquired pneumonia secondary to an opportunistic infection. The organism is susceptible to most antibiotics including piperacillin/tazobactam, and infected patients have shown good outcomes with empirical antibiotic treatment. The organism can, however, acquire plasmid-borne antibiotic resistance genes, including extended-spectrum β-lactamase and carbapenemase genes, which may result in severe and often fatal infections [7,8]. Our patient did not receive antibiotics from either of these categories of drugs.

## Conclusions

*R. planticola* is a relatively newly described pathogen, with its incidence and description increasing in recent years. Minimal literature has been published regarding its characteristics and presentations in the infected individual; so, this case report highlights one of the very rare occasions where it has been implicated in the diagnosis of bacterial pneumonia and a subsequent myasthenic crisis. Its tendency to offer genetic resistance to certain antibiotics also makes it highly important to be closely monitored and studied. Further information and literature on the topic would prove to be of value.
